# Knowledge, attitude, and use of mHealth technology among students in Ghana: A university-based survey

**DOI:** 10.1186/s12911-019-0947-0

**Published:** 2019-11-12

**Authors:** Prince Peprah, Emmanuel Mawuli Abalo, Williams Agyemang-Duah, Razak M Gyasi, Okwei Reforce, Julius Nyonyo, Godfred Amankwaa, Jones Amoako, Paulinus Kaaratoore

**Affiliations:** 1 Department of Geography and Rural Development, Kwame Nkrumah University of Science and Technology, Kumasi, Ghana; 2 African Population and Health Research Center, Nairobi, Kenya

**Keywords:** mHealth, Knowledge, Utilization, University students, KNUST, Ghana, Integration

## Abstract

**Background:**

Interest in mHealth interventions, defined as the use of mobile phones to access healthcare is increasingly becoming popular globally. Given its technology-based applications, university students may be key clients of the mHealth adoption but studies are rare in sub-Saharan Africa. This study provides a snapshot and baseline evidence on knowledge, attitude and use of mHealth among university students in Ghana.

**Methods:**

Using a self-administered questionnaire, we collected data between April and June 2017 from 963 randomly sampled undergraduate students at the Kwame Nkrumah University of Science and Technology (KNUST). Pearson’s Chi-square (*χ*^2^) test assessed the differences between variables whilst  logistic regression models estimated the independent predictors of use of mHealth with *p* < 0.05 as significant.

**Results:**

Knowledge on mHealth was moderately high. Specifically, more than half of the sample reported awareness of mHealth although the prevalence of use of mHealth stood at 51%. Logistic regressions revealed that mHealth use was positively associated with respondents’ year (second year: OR = 1.704, 95% CI: 1.185–2.452, and third year: OR = 1.528, 95% CI: 1.060–2.202), and monthly income (OR:3.112, 95%CI: 1.180-8.211). However, ethnicity [(OR = 0.761, 95% CI (0.580–0.997)] was negatively associated with the use of mHealth technology.

**Conclusion:**

Findings suggest that knowledge of mHealth among university students is low. Policy and public health interventions for urgent awareness creation and promotion of use of mHealth as well as its possible integration into the mainstream healthcare system in Ghana are timely.

## Background

Use of mobile phones (mHealth) to access healthcare including treatment, emergency medical response and education is gaining attention worldwide as a complementary strategy for strengthening health systems emphasizing the role of current environmental and technological improvements in the lives of people [1]. Information communication technology (ICT) has become the main social process used to deliver health care and to elevate public health [2]. Mobile phones and the internet are growing rapidly in the low-and middle-income countries (LMICs) and, has been recognized as powerful tools for improving efficiency in the health sector [3]. Amongst analysts of global health, there is growing enthusiasm for the possibilities opened up by these technologies, specifically the rapid spread of mobile phone coverage which includes substantial increasing access to health-related information, and advice and expert medical consultations [4]. Consequently, researchers indicate that we are reaching a ‘tipping point’ in the organisation of health systems in which new technology will drive new organisational arrangements [5].

Worldwide, so many mHealth projects are being implemented for healthcare delivery, disease surveillance, health education and health promotion behaviour change communication, and training of the health workforce [6–9]. Indeed, mHealth has emerged as a viable solution for wide-ranging challenges in healthcare delivery in LMICs, including Ghana as a result of high community mobile phone penetration and a shortage in the health workforce, [10, 11].

In recent time, most promising alternate surveillance systems for the enhancement of self-care practices in individuals across a broad field in the world, including diabetes [12], cancer [13], coronary heart diseases [14], anxiety disorder [15] eating disorders [16], substance use [17, 18], are those based on mobile technologies. Significantly, there is improved communication as a result of the invention and usage of mobile phones. Additionally, there is also a growing global trend in harnessing this technology for behavioural change, disease surveillance, prevention and control [19] as well as promoting health-related attitudes and behaviours such as weight reduction [20], physical activity [21] and smoking cessation [22, 23]. Reasonably large, interventions delivered through mobile phones have the tendency of empowering service users with greater choice and control over their health care needs [24].

With reference to recent estimates by National Communication Authority, approximately 20 million phones were used across Ghana, overtaking that of Switzerland and other developed countries [25]. Despite the proliferation of mobile phones and increasing use of the technology for various purposes in Ghana, the specific knowledge and utilisation of mobile phones for health and associated correlates are less understood. In addition, mobile phones usage for accessing healthcare in Ghana has been rarely evaluated, limiting evidence-based policy adoption. As a result of the increasing use of mobile phones in Ghana, it is critical to understand mHealth knowledge base and utilisation amongst Ghanaians. This cross-sectional study aims to contribute to the understanding of knowledge and the use of mHealth technology among university students in Ghana. Such a knowledge from the study could help policy dialogue, not only in Ghana, but in other LMICs globally. Investigation of the knowledge and utilisation of mHealth will help offer appropriate policy recommendations for improving and strengthening programmes directed at enhancing effective and efficient mHealth use.

## Methods

### Design and context

The present campus-based cross-sectional study was carried out among undergraduate students in Kwame Nkrumah University of Science and Technology (KNUST), Ghana. Located in Kumasi, the university is situated approximately on 16 square-kilometres, about seven kilometres away from the central business district of Kumasi. KNUST as of July 2018 had a student population of 42,590, of which 36,807 constitute undergraduates including international students [26]. The institution attracts people from all parts of the country and other neighbouring countries with different socio-cultural traits who have the capacity to bring to bear different experiences, attitudes and knowledge on mHealth. Conducting this study at KNUST is envisaged to provide specific information that could be extrapolated to serve as a measure to determine mHealth knowledge and utilisation among the general population given that universities often serve as a bridge between the academic world and the society.

### Sample and procedure

Owing to the number of colleges, diverse population characteristics, and socio-economic backgrounds of students at the university, health and non-health students were included in this study to give fair representation, using a two-phase cluster and simple random sampling methods. One department was selected from each of the six colleges after clustering the colleges into health and non-health per the university definition and then using a simple random sampling technique. All health departments were listed and labelled ‘health’, while all non-health departments were marked as ‘non-health’ purposely for easy identification. The process was followed by random selection of three departments to represent each of the health-inclined (Biological Science, Pharmacy and Nursing) and non-health departments (Geography and Rural Development, Economics, and Sociology and Social Work). The study considered individual undergraduate students at all levels; from Levels 100 to 400, across all age and gender categories. Out of approximately 36,807 undergraduates, this study randomly selected 1003 students, taking into consideration the programmes of study. The sample was distributed to the programmes of study using population size as a measure. In this respect, 414 non-health-related students (including Geography and Rural Development, Economics, and Sociology and Social Work) and 589 health students (including Biological Science, Pharmacy, and Nursing) were enrolled.

Data collection spanned between April and June 2017, using a 63-itemised self-administered questionnaire developed by the first author in consultation with all co-authors based on an extensive literature review. The questionnaire included the following components: 1) background information; 2) perceived effectiveness and necessity of mHealth technology variables; 3) conditions for which mHealth were used. The focus of the questionnaire sought to understand the knowledge, attitude and utilisation of mHealth among university students. To ensure quality control, a close monitoring coupled with spot-checks and re-checks on completed questionnaires were done by the researchers. The completion of each questionnaire took about 30 min on average. All study respondents were initially briefed on the main purpose of this study and all that was required of them. Participation was voluntary. Informed written and verbal consents were obtained from all research respondents. Respondents were assured of strict confidentiality and anonymity of the responses they provided. To protect the identity of the respondents, no names or other identifying features of the respondents were collected.

The questionnaires were distributed to the study respondents during their normal lecture periods. For a better understanding, various items were explained to students (respondents) by trained field research assistants recruited from the Department of Geography and Rural Development, KNUST. The authors explained the content of the questionnaire to the trained field assistants before distributing it to the various classes. Moreover, an author followed these participants to the individual classes to help clarify any ambiguity regarding the interpretation and understanding of the questionnaire by the students. To help check call-back challenges, the distribution, completion and collection of questionnaires were done by hand and in the same day. This provided the avenue to improve on the response rate of participation. Data collection processes were closely monitored by the researchers.

### Measures

#### Outcome variable

Use of mHealth technology was measured as “non-use or use of mHealth technology during the last 12 months ahead of the survey”. In addition, mHealth knowledge was determined as “aware or unaware of mHealth technology”. mHealth was defined as the use of mobile phones to assess healthcare including treatment, service, emergency medical response and education [7]. This definition was provided to give the respondents a better understanding of the term mHealth in order to aid correct and adequate answers to questions used to assess the outcome variable: “Have you ever used mobile phone to assess healthcare or for any health information within the last 12 months?”, “Are you aware that mobile phones can be used to access healthcare?” The questions were binary measures and the outcomes were entered as 0 = no or 1 = yes.

#### Exposure variables

Various demographic and socio-economic variables, perceived health-related belief variables and health status and conditions for which mHealth was used were considered. In this study, the main explanatory variables were knowledge and utilisation of mHealth. Knowledge and utilisation were categorised as yes =1  or no = 0 . Other demographic, socio-economic and perceived health-related variables measured included: age (in years), department (health related and non-health related) (Geography and rural development, Economics, Sociology and Social work, Nursing, Biological Science and Pharmacy) residence (on-campus/off-campus), level of study (100/ 200/ 300/ 400), income status (in Ghana Cedi (GH¢)), ethnicity (Akan/ Ewe/ Ga-Dangme/ Mole-Dagbani/ Guan/ Gurma), religious affiliation (Christianity/ Islam/ Traditional African Religion/ others), insurance status (insured/uninsured) and other attitudinal issues concerning mHealth. Effectiveness and necessity of mHealth was validated using a four Likert scaled response which was added to the questionnaire after the pre-test with a section of the students. Effectiveness as used in this study refers to the ease and ability of respondents to access adequate health services from these mobile platforms whereas necessity connotes its importance within the healthcare seeking and accessibility space in Ghana.

### Statistical analysis

The data were analyzed using the Predictive Analytics Software (PASW) for Windows application programme (version 17.0). A probability value (*p*) less than 5% was considered statistically significant [27]. Descriptive statistics were used to describe the study sample . Differences in counts and proportions between variables were assessed for the following: awareness of use of mobile phones for accessing healthcare by gender and programme of study, prevalence and pattern of mHealth use among students by gender and students’ attitudes to and perception about mHealth, using Chi-square (*χ*^2^) test analysis. Predictions of mHealth knowledge and use were estimated by logistic regression models in which the use of mHealth was regressed on the sociodemographic characteristics of the respondents. Odds ratios and the corresponding 95% confidence intervals(CI) were calculated and presented. Diagnostic statistic tool, Hosmer-Lemeshow, was used to test the goodness of fit and the strength of the  regression models.

## Result

### Sample demographic profile

A total of 963 questionnaires were returned, excluding all those that were not completely filled representing a response rate of 96%. Table 1 presents the background characteristics of the respondents. Majority of the study respondents were males (518, 53.8%), aged 21- 23 years (456, 47.3%), pursue health related courses (589, 61.2%) and in the first year (308, 32%). More than half of the respondents resided outside campus (535, 55.6%), were from the Akan Ethnic group (520, 54%) and also professed to Christian faith (701, 72.8%). Most of the respondents were from economically well-off families (445, 46.2%) and received an estimated monthly imbursement of not more than GH¢ 300 (513, 53.3%). Whereas all the study respondents owned a mobile phone, about 88% of these were smartphones.We observed a statistically significant difference between males and females for all the study variables apart from place of residence and faith professed  (*p* < 0.05) (see Table 1).

**Table 1:** Background characteristics of the study respondents

Variable	Response	Male	Female	*p-value*
		*n* = 518 (%)	*n* = 455 (%)	
Age	18–20	175 (33.8)	191 (42.9)	.001*
	21–23	247 (47.7)	209 (47.0)	
	at least 24	96 (18.5)	45 (10.1)	
Department	Non-health programmes^a^	188 (36.3)	186 (41.8)	.044*
	Health programmes^b^	330 (63.7)	259 (58.2)	
Year of study	1st	141 (27.2)	167 (37.5)	.001*
	2nd	136 (26.2)	75 (16.8)	
	3rd	177 (34.2)	143 (32.2)	
	4th	64 (12.4)	60 (13.5)	
Place of residence	on campus	217 (41.9)	211 (47.4)	0.694
	off campus	301 (58.1)	234 (52.6)	
Ethnicity	Akan	246 (47.5)	274 (61.6)	.001*
	Other Ethnic Groups	272 (52.5)	171 (38.4)	
Faith professed	Christianity	375 (73.2)	326 (73.4)	.829
	Islam	109 (21.1)	94 (21.1)	
	African traditional religion	34 (6.6)	25 (5.6)	
Estimated monthly pocket money	at most 300 Cedis	268 (51.7)	245 (55.1)	.014*
	301–400 Cedis	143 (27.6)	119 (26.7)	
	401–500 Cedis	50 (9.6)	57 (12.8)	
	501–600 Cedis	26 (5.0)	16 (3.6)	
	601–700	17 (3.3)	4 (0.9)	
	at least 701	14 (2.8)	4 (0.9)	
Family’s socioeconomic status	quite poor	102 (19.7)	65 (14.6)	.001*
	not very well off	189 (36.5)	105 (23.6)	
	quite well off	203 (39.2)	242 (54.4)	
	wealthy	24 (4.6)	33 (7.4)	
Smartphone Ownership	Yes	475 (91.7)	374 (84)	.001*
	No	43 (8.3)	71 (16)	

*p<  0.05

^a^Geography and Rural Development, Economics and Sociology and Social Work

^b^Biological Science, Pharmacy and Nursing

### Awareness of use of mobile phones for accessing healthcare by gender and Programme of study

Table 2 presents respondents’ knowledge on the use of mobile phones for accessing healthcare information based on gender and programme of study . Overall, (684, 71%) respondents had knowledge about the use of mobile phones for healthcare. Specifically, more males (350, 51%) than females (334, 49%) were aware of the use of mobile phones for accessing healthcare. Results revealed statistically significant difference between genders  in awareness of use of mobile phones for accessing healthcare information (53.8% vs. 46.2%; *p* < 0.011). Regarding respondents’ knowledge on the use of mHealth by programme, 274 (40%) those reading non-health programmeswere more likely to be aware of mHealth compared to those reading health-related programmes but difference did not reach significance.

**Table 2:** Knowledge of use of mobile phones for healthcare by gender and programmes

Variables	Responses	Knowledge on use of mobile phones for accessing health information			*p-value*
		Yes	No	Total	
		*N* (%)	*N* (%)	*N* (%)	
Gender	Male	350 (51.2)	168 (60.2)	518 (53.8)	.011^*^
	Female	334 (48.8)	111 (39.8)	445 (46.2)	
	Total	684 (71)	279 (29)	963 (100)	
Programmes	Non-health programmes	274 (40.1)	100 (35.8)	374 (38.8)	.223
	Health programmes	410 (59.9)	179 (64.2)	589 (61.2)	
	Total	684 (71)	279 (29)	963 (100)	

*statistically significant at *p<*  0.05

### Prevalence and pattern of mHealth use among students by gender

Table 3 presents the prevalence and patterns of mHealth use by gender. Some 301 (44%) of the respondents reported use of mHealth in the last 12 months preceding the survey. About 508 (74%) of the respondents used mHealth at irregular intervals (at least once a day or at least a week and at least once a month/at least once in 3 months). About 395 (58%) of the respondents used mobile phones for accessing primary healthcare service such as diagnosis, health advice and treatment of diseases and/or sickness whereas the remaining 289 (42%) used mobile phones for general health education or information. Facebook was identified as the main platform (208, 30%) where information on health was accessed. Notwithstanding, YouTube (192, 28%), WhatsApp (114, 17%) and Twitter (79, 11%) were other platforms used by the respondents for accessing health information. Moreover, the study respondents often received healthcare information via SMS (491, 72%) and direct consultation over mobile phone (193, 28%). These differences were  statistically significant between genders and other platforms used for accessing mHealth information (*p* = 0.001).

**Table 3:** Use of mobile phones for health by gender

Variable	Responses	Gender			
		Male	Female	Total	*p-value*
		*n* = 350 (%)	*n* = 334 (%)	*n* = 684 (%)	
Use of mHealth in the past 12 months	No	203 (58)	180 (53.9)	383 (56)	.279
	Yes	147 (42)	154 (46.1)	301 (44)	
Frequency of mHealth use	many times, a day	93 (26.6)	83 (24.8)	176 (25.7)	.351
	at least once a day	60 (17.1)	45 (13.5)	105 (15.3)	
	at least a week	77 (22)	72 (21.6)	149 (21.8)	
	at least once a month/at least once in three months	120 (34.3)	134 (40.1)	254 (37.1)	
Type of health service sought	Primary healthcare service (diagnosis, health advice and treatment)	209 (59.7)	186 (55.7)	395 (57.7)	.159
	Health education/information	141 (40.3)	148 (44.3)	289 (42.2)	
mHealth service use	Text messages	255 (72.9)	236 (70.7)	491 (71.8)	.523
	Direct consultation over mobile phone	95 (27.1)	98 (29.3)	193 (28.2)	
Other platforms for accessing Health information	Twitter	57 (16.3)	22 (6.6)	79 (11.5)	
	Facebook	111 (31.7)	97 (29)	208 (30.4)	.001*
	WhatsApp	57 (16.3)	57 (17.1)	114 (16.7)	
	snapchat	31 (8.9)	11 (3.3)	42 (6.1)	
	Instagram	20 (5.7)	29 (8.8)	49 (7.2)	
	YouTube	74 (21.1)	118 (35.3)	192 (28.1)	

*p < 0.05

In Table 4, ascertaining the relationship between mHealth use among the respondents by programme, 301 (44%) of the respondents from both the health and non-health programme reported use of mHealth in the last 12 months preceding the survey (*p* =0 .313). Though most of the respondents (508, 74%) irregularly (at least once a day or at least a week and at least once a month/at least once in 3 months) used mHealth for surfing health related information (*p =0 .001*), about 289 (42%) used mobile phones for surfing healthcare information or education (*p* = 0.025). Besides browsing the internet for information on mHealth, respondents often receive healthcare information via SMSs (491, 71.8%) and direct consultation over mobile phone (193, 28.2%). Just as identified among the gender divide, Facebook (208, 30%) was the main platform respondents used to access healthcare information, besides YouTube (192, 28%), WhatsApp (114, 17%) and Twitter (79, 11%). These discoveries showed statistically significant differences among the non-health and health programmes (*p* =0 .001) from Pearson’s Chi-square analysis conducted.

**Table 4:** Use of mobile phone for health by programme (health and non-health)

Variable	Responses	Department			
		Non-health Programmes	Health Programmes	Total	*p-value*
		*n* = 274 (%)	*n* = 410 (%)	*n* = 684 (%)	
Use of mHealth in the past 12 months	No	147 (53.6)	236 (57.6)	383 (56)	.313
	Yes	127 (46.3)	174 (42.4)	301 (44)	
Frequency of mHealth use	many times, a day	64 (23.4)	112 (27.3)	176 (25.7)	
	at least once a day	18 (6.6)	87 (21.2)	105 (15.3)	.001*
	at least a week	62 (22.6)	87 (21.2)	149 (21.8)	
	at least once a month/at least once in three months	130 (47.4)	124 (30.2)	254 (37.1)	
Health information sought	Primary healthcare service (diagnosis, health advice and treatment)	142 (51.8)	253 (61.7)	395 (57.7)	.025*
	Health education/information	132 (48.2)	157 (38.3)	289 (42.2)	
Source of mHealth information	Twitter	17 (6.2)	62 (15.1)	79 (11.5)	.001*
	Facebook	73 (26.6)	135 (32.9)	208 (30.4)	
	WhatsApp	51 (18.6)	63 (15.4)	114 (16.7)	
	snapchat	20 (7.3)	22 (5.4)	42 (6.1)	
	Instagram	22 (8)	27 (6.6)	49 (7.2)	
	YouTube	91 (33.2)	101 (24.6)	192 (28.1)	
mHealth service use	Text messages	196 (71.5)	295 (71.9)	491 (71.8)	.905
	Direct consultation over mobile phone	78 (28.5)	115 (28)	193 (28.2)	

*statistically significant at *p* <0.05

### Predictors for utilization of mHealth

From the model summary in Table 5, only 4.5% of the independent variables explains variation in the dependent variable. Regressions showed that being in second year (OR = 1.704, 95% CI: 1.185–2.452) and third year (OR = 1.528, 95% CI: 1.060–2.202) were more likely to use mHealth compared with those in first year. Meanwhile, respondents from other ethnicities were less likely to use mHealth in relation to those from the Akan ethnic groups (OR=0.761, 95%CI:0.580-0.997). Further, respondents who received an estimated monthly income of Gh¢ 601 and 700 were more likely to r use mHealth in the last 12 months as compared to those who received at most Gh¢ 300. Thus, affluence, ethnicity and class of respondents were indepedent predictors of mHealth use among university students.

**Table 5:** Binary regression analysis of utilization of mobile phones for healthcare

non-use or use of mobile phones for health care during the last 12 months ahead of the survey: Yes/No				
		OR	95% CI	*p-value*
Department	Non-health programmes	1		
	Health programmes	1.008	0.734–1.384	.962
Year of study	1st	1		
	2nd	1.704	1.185–2.452	.004*
	3rd	1.528	1.060–2.202	.023*
	4th	1.266	0.804–1.994	.309
Ethnicity	Akan	1		
	Other Ethnic Groups	0.761	0.580–0.997	.047*
Current religious affiliation	Christianity	1		
	Islam	0.900	0.653–1.242	.523
	African traditional religion	0.974	0.563–1.688	.927
Estimated average monthly pocket money	at most Gh¢ 300	1		
	Gh¢ 301–400	1.308	0.965–1.773	.084
	Gh¢ 401–500	0.840	0.546–1.293	.428
	Gh¢ 501–600	0.582	0.291–1.166	.127
	Gh¢ 601–700	3.112	1.180–8.211	.022*
	at least Gh¢ 701	0.369	0.118–1.152	.086

*p < 0 .05; OR Odds ratio, *CI* Confidence interval; Nagelkerke *R* = 0.045; Hosmer-Lemeshow 1.179

### Attitudes and perception of mHealth

Generally, the use of mobile phone for accessing health information was found to be effective based on respondents’ self-report (very effective/effective; 321:99%). Respondents agreed that using mobile phone for health information offered greater security (strongly agree/agree; 383:56%), and 72% believed accessing mHealth information via smartphones increases information dissemination among friends and colleagues (strongly agree/agree; 490:71.6%). A significant percentage of the respondents appraised the convenience associated with mHealth information in comparison to other sources (strongly agree/agree; 446:65.2%). Respondents’ attitude towards, and perception about the effectiveness of mHealth, decreased with increasing class level (*p<* 0.05). Besides the convenience associated with mHealth (*p = 0.*045), the remaining variables were not statistically significant (*p* < 0.05) (See Table 6).

**Table 6:** Respondents’ attitudes to and perception of mobile phones for healthcare

Variable	Responses	Level of Students					
		1st	2nd	3rd	4th	Total	
		*N* (%)	*N* (%)	*N* (%)	*N* (%)	*N* (%)	*p-value*
Effectiveness of using mobile phone for accessing healthcare	Very Effective	55 (48.2)	32 (50)	34 (34)	22 (47.8)	143 (44.1)	
	Effective	59 (51.7)	32 (50)	64 (64)	23 (50)	178 (54.9)	
	Not Effective	0 (0)	0 (0)	2 (2)	1 (2.2)	3 (0.9)	0.159
Using mobile phone to access healthcare offers greater security	Strongly Agree	38 (17)	26 (18)	32 (14.6)	14 (14.4)	110 (16.1)	
	Agree	102 (45.5)	54 (37.5)	80 (36.5)	37 (38.1)	273 (39.9)	
	Not sure	70 (31.2)	40 (27.8)	79 (36.1)	36 (37.1)	225 (32.9)	0.164
	Disagree	11 (4.9)	20 (13.9)	21 (9.6)	7 (7.2)	59 (8.6)	
	Strongly Disagree	3 (1.3)	4 (2.8)	7 (3.2)	3 (3.1)	17 (2.5)	
Easy to share mobile-phone-based medical information	Strongly Agree	48 (21.4)	42 (29.2)	54 (24.7)	28 (28.9)	172 (25.1)	
	Agree	111 (49.5)	67 (46.5)	95 (43.4)	45 (46.4)	318 (46.5)	0.253
	Not sure	58 (25.9)	30 (20.8)	53 (24.2)	21 (21.6)	162 (23.7)	
	Disagree	7 (3.1)	5 (3.5)	17 (7.8)	3 (3.1)	32 (4.7)	
Using mobile phone to access healthcare is convenient	Strongly Agree	56 (25)	26 (18.1)	45 (20.5)	18 (18.6)	145 (21.2)	
	Agree	97 (43.3)	72 (50)	88 (40.2)	44 (45.4)	301 (44)	
	Not sure	53 (23.7)	28 (19.4)	57 (26)	24 (24.7)	162 (23.7)	0.045^*^
	Disagree	10 (4.5)	13 (9)	28 (12.8)	7 (7.2)	58 (8.5)	
	Strongly Disagree	8 (3.6)	5 (3.5)	1 (0.5)	4 (4.1)	18 (2.6)	

*p < 0.05

## Discussion

This study teases out insight into access, knowledge and utilisation of mobile phones for healthcare among undergraduate students in KNUST, Ghana. This study is novel in that it provides the first campus-based evidence on mHealth knowledge and utilisation among students. The present study revealed, across different programmes of study at all academic levels and genders, a high level of mobile phone possession among students, perhaps due to the urban setting of the university, which is in line with the national scale of high cell and smartphones penetration and possession among the general populace in Ghana. Ownership and possession of mobile phones among the population and healthcare providers is the most important factor in supporting large scale implementation of effective and efficient mobile based healthcare intervention [28–30] . Ghana used to have six telecommunication networks/providers in the mobile phone arena; MTN, Vodafone, Glo, Airtel, Tigo and Expresso [25]. However, the merging of Airtel and Tigo as a single entity, Airtel-Tigo, has reduced the total number to five. These mobile networks and the associated subscriptions offer a unique opportunity for lessening the various barriers to healthcare delivery and health promotion in Ghana through mobile-based health interventions. This high mobile phone use prevalence suggests a possible readiness of mHealth adoption on the part of the general population as they meet the first requirement for mHealth implementation.

In this study, the high possession of mobile phones among the respondents and penetration of mobile phones on campus reflected in optimal knowledge and awareness on the use of smartphones for accessing health information among students. This contradicts studies in other developing country like Bangladesh by Khatun et al., where mHealth awareness was low among respondents [30]. However, previous studies reporting low knowledge of mHealth among respondents were mainly conducted in rural communities where access to mobile phones and knowledge of mobile phone operations were limited compared to a university campus, where mobile and smartphones have proliferated. By inference, mHealth technology can be said to be an emerging technology which requires deliberate efforts for widespread awareness, knowledge and use across the general population to enhance possible acceptance and adoption in Ghana.

In consistent with other previous studies in Bangladesh [30] and Kenya [31], one important finding was that there was a significant difference in mHealth knowledge among male and female respondents. This study found gender inequity of mHealth knowledge as female students lag behind in knowledge of mHealth. More males were privy to the use of mobile phones for accessing healthcare than their female counterparts. The study observed gender inequalities in the use of mHealth has the propensity of hindering mHealth programmes targeting females including Reproductive Health, Maternal Newborn and Child Health (MNCH), and childhood immunization programmes [30]. By inference, this could serve as a sociocultural barrier to potential mHealth innovation adoption, implementation and use in Ghana when this low knowledge of mHealth among the respondents is not addressed for maximum utilisation of the potentials of mHealth across genders.

The present study demonstrated a relatively moderate prevalence of use of mHealth technology among male and female students as well as health and non-health students in KNUST. The optimal knowledge and awareness on the use of mHealth among the study respondents did not necessarily correspond to optimal use of mHealth in the last 12 months preceding the survey, while students who used mHealth used it irregularly. This low prevalence of mHealth use has been reported in other studies in Africa countries such as Benin [32] and Nigeria [33]. By implication, subscription and ownership of mobile phones indicators are not good proxy measures of the value of the technology, since they do not indicate the range of purposes for which different population groups are using phones and do not provide any means of assessing the benefits resulting from that use [34]. With this finding, it can be argued that the use of mobile phones for healthcare in Ghana is not developed and calls for efforts to ensure intensified education and sensitisation about mHealth use. However, in this study, being a health student was found to be associated with the frequency of mHealth use as the study established a significant difference in use of mHealth by programme of study. The health-related students, with reference to the health and the aligned health science programmes, one way or the other have a direct connection with the new emerging technology in healthcare system. These students, because of their training/learning orientations, may influence the extent to which they access and use healthcare through mobile phones. Mobile telecommunication in Ghana is currently engaged in public activities such as transaction services including mobile money transfers and other mobile payments. The current successful and growing mobile phone banking sector suggests clearly that similar intervention involving mHealth sector could be developed and integrated into the national healthcare system where quality and timely health needs would be prioritised. Inasmuch as they are engaged in such activities, they can also serve as a platform for health intervention of any kind where mobile phone-based health alerts can take place since they already have similar platforms in other sectors [32]. The innovation, when properly integrated, can be used to establish surveillance systems through the network of healthcare extension workers where they could be provided with a number of specific diseases needing immediate reporting in human and animals [35]. It could also be used to provide healthcare information to the general public by alerting people during emergency situations and outbreaks. Mobile phone-based platforms can also be used for medication reminders, adherence and defaulter tracing. Furthermore, mobile phones could be used as tools for strengthening the current health management information system to facilitate collection and compilation of information from wide areas in Ghana. As a university with busy lecture and assignment schedules among students, lecturers and other workers, adopting and integrating mHealth technology into the campus healthcare system will ease patients with long period of hours spent and other inconveniences encountered at students’ clinics and hospitals at the campus. Also, mobile technology-based healthcare system will be useful in providing near to real-time data, with the potential for enhancing timely response, especially during emergency cases on campus [31]. Harnessing such a highly effective mobile technology platforms for reporting directly health issues to workers as well as seeking treatment by oneself or from health professional is considered to allow rapid data access, use, and quality assurance by stakeholders at local and national levels.

Not surprisingly, the present study provided evidence to suggest that platforms where students often access mHealth information on their phones were Facebook, Snapchat, Instagram, Youtube, WhatsApp, and Twitter, compared to the widely reported phone calls and personalised SMS interventions [9, 11, 33, 36–38]. Considering this finding, students access personal health care and information with mobile phones from these platforms without direct contact (face-to-face consultation) with healthcare providers. This suggests that users of mHealth are replacing direct consultation with providers with indirect consultation through surfing health information online with the use of software applications on phones. In a university environment where technology is advanced, the use of these digitised platforms for healthcare is not novel. The availability of free internet services across university campuses have improved uptake of software applications by students for health care and information through mobile phones. However, the concern is that these preferred methods and platforms for health care and information among students outside such a technological environment will not be practical and effective, particularly due to technological disparities across Ghana. In other communities, both urban and rural, different mHealth programmes such as phone calls and SMS which are easy, reliable and cost-effective may prove successful considering the rate of mobile phone usage and telecommunication reach out.

Importantly, this study has provided evidence to demonstrate that certain demographic and socioeconomic positions play a major role in mHealth use decisions. In the logistic regression model with mHealth use in the last 12 months as the dependent variable, our study established that students’ socio-demographic characteristics, specifically ethnicity, class of respondents and high average monthly income, predicted mHealth use with significance. Other demographic characteristics such as religious affiliation and department insignificantly predicted mHealth use thus showing the former as better functions of mHealth use which should be targeted by policymakers in strengthening the use of then platform in future policies. Although mHealth use was frequently accessed among students in the health-related programmes in Table 5, it did not predict with significance, mHealth utilisation in the last 12 months (Table 6). This could mean that, differences in the frequency of mHealth use between the two study programmes does not necessarily translate into a strong predictor of mHealth use. At best, policies targeted at streamlining the use of mHealth in university institutions should not only consider the programme of study, but also other salient variables such as students’ ethnicity, year of study across health and non-health programmes and their estimated monthly income: socio-demographic characteristics of the target population. It can, therefore, be argued that mHealth technology use is associated with user’s socio-economic characteristics which have been previously reported [30].

Significantly, key issues regarding students’ attitude to and perception about mHealth among students were observed. The study found that students have evaluated the services of mHealth and are able to rate its effectiveness, convenience, safety and information sharing capacity. The study revealed that students evaluated mHealth to be convenient for accessing healthcare information which was statistically significant. Also, most of the students perceived mHealth to offer a sense of security, effectiveness, and enhance information sharing among colleagues and relatives. These positive attitudes to and perceptions about mHealth among students reflect the positive outcomes they receive.

To the best of our knowledge, this is the first known campus-based study around insights into knowledge and utilisation of mHealth in Ghana. By combining perspectives of students from different departments including health and non-health programmes, the study findings have diverse advantages. Our findings will inform programmers and implementers to design future mHealth and implement programmes to promote knowledge of mHealth with reference to the actual and potential benefits of the innovation. It will also instigate appropriate mHealth initiatives that can reach, and are usable by students, especially women and the poor, when integrated into the national healthcare system.

Some limitations of this study include its basis on a cross-sectional survey design in a university campus, which is limited to the available mHealth services in the university. Other settings outside the university community may have different contexts such as greater availability and awareness of mHealth services. Second, the measures were derived from self-reports of respondents of mHealth use, thereby exposing the findings to potential response bias and social desirability bias. Also, the study did not include investigation of type of health information sought by users of mHealth and barriers to mHealth use and this calls for further studies to ascertain evidence in these areas.

## Conclusion

The study provides evidence to demonstrate that mHealth knowledge differs between males and females. The study revealed a moderate prevalence of use of mHealth among undergraduate students in KNUST, Ghana. To a larger extent, different socio-demographic factors were associated with students’ use of mHealth. Given the upsurge implementation coupled with the numerous benefits associated with mHealth, these findings can help ensure effective public health policy. Integration of mHealth programme in the university’s healthcare system may prevent untimely emergency cases of disease outbreaks on campus especially with the prevalence of sexually transmitted diseases in recent times on campus. Such an integrative process should be a collaborative one between all stakeholders, including the Ministry of Health and mobile telecommunication providers in Ghana, to create win-win partnerships to tapping the potential benefits of high mobile phone prevalence.

## Data Availability

The datasets used and/or analysed during the current study are available from the corresponding author on reasonable request.

## Abbreviations

mHealth: Mobile Health
KNUST: Kwame Nkrumah University of Science and Technology
ICT: Information communication technology
PASW: Predictive Analytics Software
MTN: Mobile Telecommunication Network
Glo: Globacom Limited
MNCH: Maternal Newborn and Child Health
